# Supplementary material for: Neuroferritinopathy Human-Induced Pluripotent Stem Cell-Derived Astrocytes Reveal an Active Role of Free Intracellular Iron in Astrocyte Reactivity
Source: Int J Mol Sci. 2025 Jun 27;26(13):6197. doi: 10.3390/ijms26136197 (PMC12249756; doi:10.3390/ijms26136197)
Supplement: Supplementary file 1 [file ijms-26-06197-s001.zip › ijms-3590581-supplementary.pdf]

Supplementary material

Supplementary Table: a Ms=mouse, Rb=rabbit, Gt=goat, Dk=dounkey
b WB=western blotting, IF= immunofluorescence

Antibody-Species [a]	Manufacturer	Catalog Number	Application [b] and Dilution
**TfR1 - Ms**	Zymed Laboratories	13-6800	WB 1:1500
**β-Actin - Ms**	Sigma-Aldrich	A5441	WB 1:5000, IF 1:800
**MDA - Rb**	Cell Biolabs	233101	WB 1:800
**GPX4 - Rb**	ABclonal	A13309	WB 1:2000
**EAAT2-Ms**	Santa Cruz	Sc-365634	IF 1:200 WB 1:500
**Fpn-Rb**	Alpha Diagnostic	MTP11-S	WB 1:1000
**DMT1-IRE-Rb**	Alpha Diagnostic	NRAMP22-S	WB 1:1000
**Mouse IgG HRP - Rb**	Sigma–Aldrich	A9044	WB 1:100000
**Rabbit IgG HRP - Gt**	Sigma–Aldrich	A9169	WB 1:100000
**GFAP - Rb**	Dako	Z0334	IF 1:250
**FtH -Ms**	Homemade	Luzzago et al 1986	ELISA 5-10μg/ml
**p62-Ms**	Santa Cruz	Sc-28359	WB 1:500
**LCN2-Rb**	Merck	SAB 1410751	WB1:800
**Nrf2-Rb**	Abcam	Ab 137550	WB 1:500
**Mouse IgG Alexa488 - Dk**	Imm. Sciences	IS20014	IF 1:800
**Mouse IgG Alexa546 - Dk**	Imm. Sciences	IS20305	IF 1:800
**Rabbit IgG Alexa488 - Dk**	Imm. Sciences	IS20015	IF 1:800
**Rabbit IgG Alexa546 - Dk**	Imm. Sciences	IS20308	IF 1:800

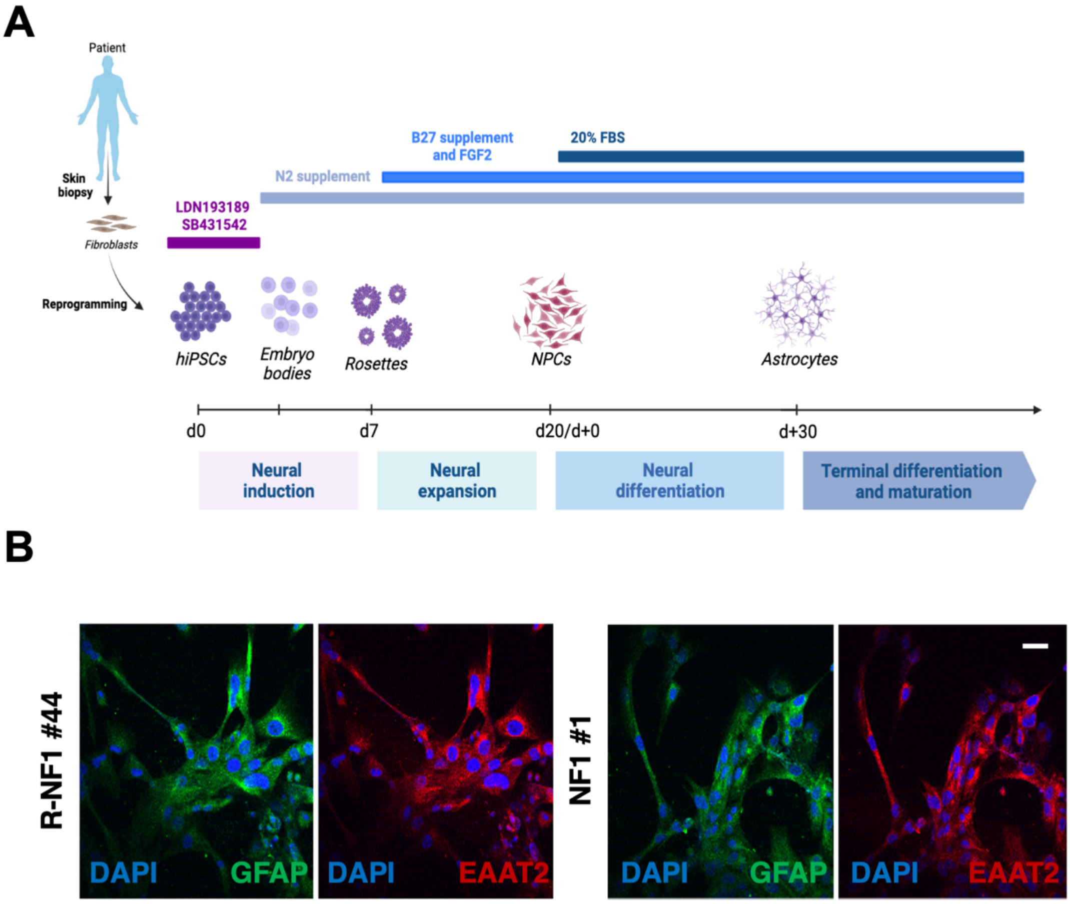

Figure S1. Development and characterization of d-astrocytes. A Graphical method representing astrocyte differentiation. NPCs were obtained by differentiating hiPSCs following a previously described method [41]. **B** Representative immunofluorescence images of d-astrocytes from one control (R-NF1) and one patient (NF1). Astrocytes were stained with specific markers for glial fibrillary acidic protein (GFAP, green) and excitatory amino acid transporter 2 (EAAT2, red). Nuclei were stained with DAPI (blue). Scale bars, 20μm.

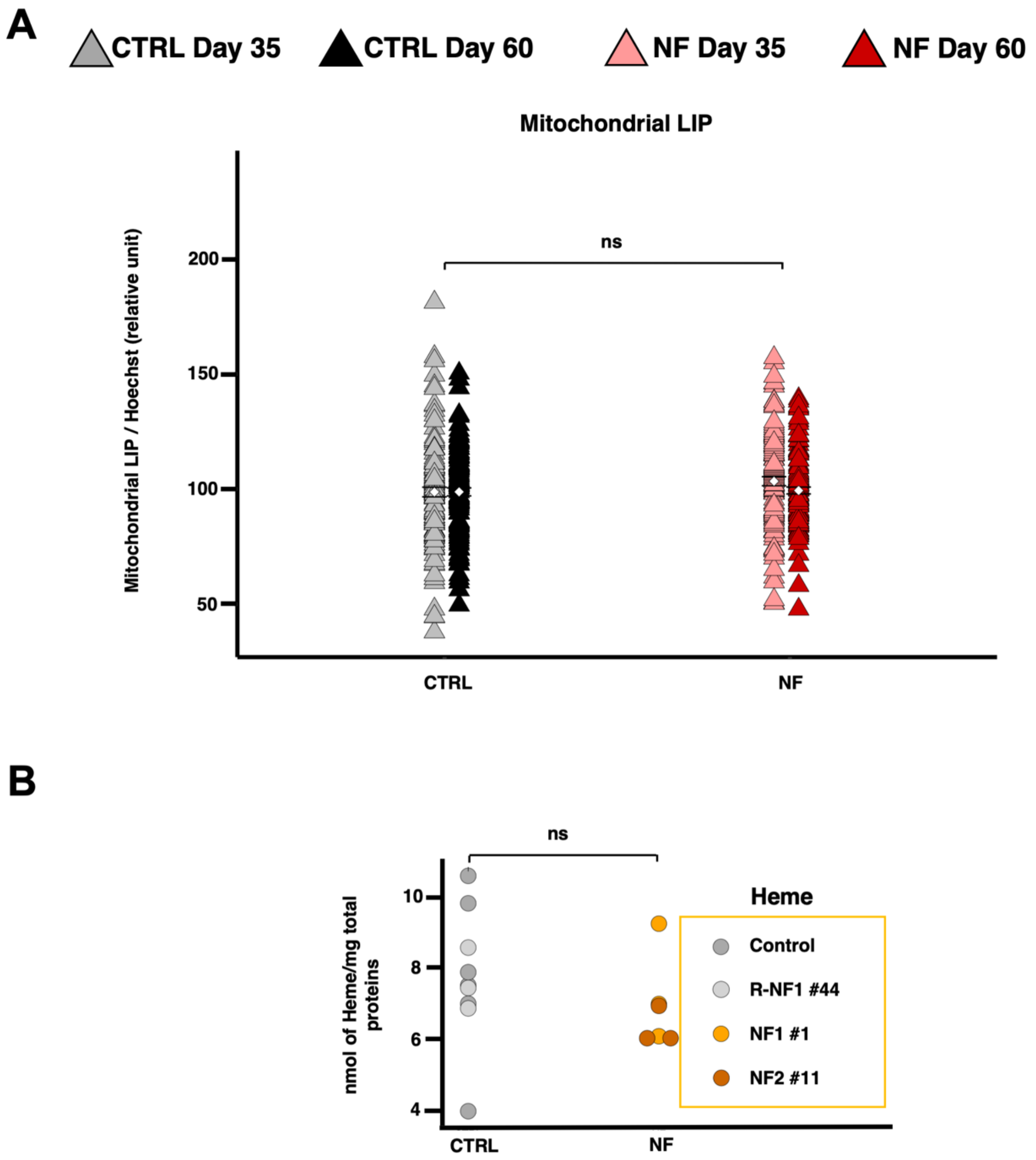

Figure S2. Mitochondrial involvement. A Evaluation of mitochondrial LIP in 35 and 60 Day d-astrocytes explored using the specific probe RPA. Gray points represent controls on Day 35 and black on Day 60, light red represents patients on Day 35, and dark red on Day 60. The graph shows results from two independent experiments (LME models, described in Materials and Methods). **B** Heme quantification in d-astrocytes by absorbance at 400 nm of the soluble cell lysates. The graph shows results from three independent experiments (LME models, described in Materials and Methods). The significance bracket indicates the statistical significance of the pathology effect.
